# Dengue Epidemiology in 7 Southeast Asian Countries: 24-Year, Retrospective, Multicountry Ecological Study

**DOI:** 10.2196/70491

**Published:** 2025-09-08

**Authors:** Shun-Long Weng, Fang-Yu Hung, Sung-Tse Li, Bo-Huang Liou, Chun-Yan Yeung, Yu-Lin Tai, Yi-Hsuan Wu, Ya-Ning Huang, Nan-Chang Chiu, Liang-Yen Lin, Hsin Chi, Chien-Yu Lin

**Affiliations:** 1 Department of Obstetrics and Gynecology, Hsinchu Municipal MacKay Children's Hospital, Hsinchu, Taiwan; 2 Department of Medicine, MacKay Medical College, New Taipei, Taiwan; 3 Department of Pediatrics, Hsinchu Municipal MacKay Children's Hospital, 28 Jiangong 2nd Rd, East District, Hsinchu, 300, Taiwan, 886 36119595; 4 Department of Infectious Disease, Hsinchu MacKay Memorial Hospital, Hsinchu, Taiwan; 5 Department of Pediatric Infectious Disease, MacKay Children's Hospital, Taipei, Taiwan; 6 Department of General Studies, National Hsinchu Girls’ Senior High School, Hsinchu, Taiwan

**Keywords:** dengue, COVID-19, epidemiology, Southeast Asia, public health

## Abstract

**Background:**

Dengue fever remains the most significant vector-borne disease in Southeast Asia, imposing a substantial burden on public health systems. Global warming and increased international mobility may exacerbate the disease’s prevalence. Furthermore, the unprecedented COVID-19 pandemic may have influenced the epidemiological patterns of dengue.

**Objective:**

This study aimed to evaluate epidemiological changes in dengue incidence in Southeast Asia.

**Methods:**

We conducted a retrospective, multicountry ecological study analyzing trends in dengue incidence in 7 Southeast Asian countries from January 2000 to December 2023. Data were extracted from official World Health Organization reports and national health department databases. Countries with data that were incomplete, inconsistent, or not publicly available were excluded from the final analysis. Annual incidence rates were analyzed, and linear trends were calculated to assess long-term patterns.

**Results:**

Epidemiological data from 7 Southeast Asian countries, comprising Thailand, Singapore, Vietnam, Malaysia, the Philippines, Cambodia, and Taiwan, were analyzed across the 24-year study period. A notable nadir in dengue cases was observed coinciding with the COVID-19 pandemic. Significant increasing trends in dengue incidence were identified in Singapore, Vietnam, Malaysia, and the Philippines (slopes: 8.243, 6.513, 8.737, and 8.172; *R*^2^ values: 0.14, 0.34, 0.345, and 0.46, respectively, all *P*<.05).

**Conclusions:**

Dengue fever continues to pose a significant public health challenge in Southeast Asia. Our analysis demonstrates a substantial increase in dengue cases in several countries over the study period. While a temporary decline was observed during the COVID-19 pandemic, a subsequent resurgence of cases highlights the persistent threat of dengue in the region. These findings underscore the critical need for sustained surveillance and innovative control strategies to mitigate the impact of dengue in Southeast Asia.

## Introduction

Dengue fever, a vector-borne disease transmitted primarily by *Aedes aegypti* and *Aedes albopictus* mosquitoes, remains a significant global health challenge, particularly in Southeast Asia [1]. This arboviral infection presents a wide spectrum of clinical manifestations, ranging from self-limiting febrile illness to severe, life-threatening complications [2]. The global burden of dengue is substantial, with an estimated 400 million infections annually, of which approximately 100 million result in symptomatic disease and 21,000 in fatalities [3,4]. Dengue transmission is closely associated with ambient temperature, and Southeast Asia remains a hyperendemic region [5]. According to a global systematic review of dengue disease burden, Southeast Asia exhibited significantly higher incidence rates than other world regions [6]. During the decade of 2001 to 2010, the region reported an estimated annual average of 2.9 million dengue cases and 5906 associated deaths [7]. The economic burden, including direct medical costs and productivity loss, along with substantial disability-adjusted life years, further underscores the considerable public health impact in this region. Despite this impact, specific antiviral treatments remain elusive, and effective vaccination strategies are not yet widely implemented, making dengue a persistent threat [8].

The dynamics of dengue transmission are intricately linked to environmental factors, making the disease particularly susceptible to the effects of global climate change. According to the World Meteorological Organization, global mean temperatures have shown a consistent upward trend over the past 2 decades, with an average increase of 0.2 °C per decade [9,10]. This warming trend has significant implications for the epidemiology of vector-borne diseases, including dengue [11,12]. Multiple studies have documented an increase in dengue prevalence correlated with rising temperatures and altered precipitation patterns, suggesting a potential exacerbation of disease burden in the context of ongoing climate change [11-16].

Concurrently, the unprecedented COVID-19 pandemic has dramatically reshaped the landscape of global health, influencing the epidemiological patterns of various infectious diseases [17]. The implementation of stringent public health measures, including lockdowns, international travel restrictions, and nonpharmaceutical interventions, led to a marked reduction in the incidence of several droplet- and contact-transmitted diseases, such as influenza and enterovirus infections. However, the easing of these restrictions has been associated with a resurgence of many infectious diseases, highlighting the complex interplay between public health interventions and disease dynamics [18].

In this context, the epidemiological trends of dengue during and after the COVID-19 pandemic remain largely uncertain. The potential impact of climate change and the disruptive effects of pandemic-related interventions create a unique scenario that warrants thorough investigation. Understanding these trends is crucial for public health planning, resource allocation, and the development of targeted prevention strategies. To address this knowledge gap, we conducted a comprehensive retrospective study examining epidemiological changes in dengue across several Southeast Asian countries from January 2000 to December 2023. The findings of this research have the potential to inform evidence-based policies for dengue control and prevention, contribute to our understanding of the complex interactions between dynamics, pandemics, and vector-borne diseases, and ultimately improve public health outcomes in Southeast Asia and beyond.

## Methods

### Study Design and Data Sources

We conducted a retrospective, multicountry ecological study analyzing trends in dengue incidence in 7 Southeast Asian countries from January 2000 to December 2023. Data on annual dengue cases were extracted from the following sources: (1) the World Health Organization (WHO) Global Health Observatory data repository [19], (2) the national centers for disease control and prevention of the respective countries, and (3) the ministry of health databases of the selected countries [19-26]. We first screened the WHO Global Health Observatory database to identify countries with available annual dengue incidence data [27]. Subsequently, we cross-validated the data with official national sources, including the websites of ministries of health or national centers for disease control. Countries with data that were incomplete, inconsistent, or not publicly available were excluded from the final analysis. In parallel, we retrieved national annual average temperature trends from publicly accessible climate databases [28]. A flowchart detailing the country selection process is provided in Multimedia Appendix 1.

### Data Collection and Processing

For each country, we compiled the annual number of reported dengue cases and population estimates to calculate incidence rates. Crude dengue incidence rates (cases per 100,000 population) were calculated by dividing the annual number of reported cases by the corresponding national population estimates, allowing for cross-country comparability. Population data were obtained from the publicly available United Nations demographic database [29]. Further standardization by age and sex was not performed, as the primary objective of this study was to assess overall national dengue incidence. In cases of data discrepancies between sources, national health authority reports were prioritized, followed by WHO data. Cross-verification between multiple sources was conducted for validation and quality control by 2 authors independently. Dengue is a notifiable communicable disease in all countries included in this study. We used Taiwan as an example, where the diagnosis of dengue is based on a combination of clinical, epidemiological, and laboratory criteria. Genetic typing is performed in specific scenarios, such as the identification of index cases in endemic regions or during outbreak investigations. Confirmed cases are further classified as either imported or locally acquired. In this study, both imported and local cases in Taiwan were included to characterize the composition of reported dengue cases. In addition, national annual average temperature data during the study period were retrieved from publicly accessible meteorological databases.

### Statistical Analysis

Statistical analyses were performed using R statistical software (version 4.1.0). Annual incidence rates per 100,000 population were calculated for each country, and epidemiological trends were visualized using the *ggplot2* package. Linear regression models were applied to assess the trends in dengue incidence over time, with the model specified as Y=β0+β1X+ϵ, where Y is the annual dengue incidence, X is the year, β0 is the intercept, β1 is the slope, and ϵ is the error term. Model parameters, including the slope and *R*^2^ values, were evaluated to determine the direction and strength of the trends. A *P* value less than .05 was considered statistically significant.

### Ethical Considerations

This study was conducted in accordance with the ethical standards of the Declaration of Helsinki [30]. Our study was approved by the institutional review board of MacKay Memorial Hospital, Taipei, Taiwan (20MMHIS140e). This study used publicly available, aggregated data and did not involve individual patient information. We adhered to the principles of ethical research practice throughout the study.

## Results

**Figure 1.**
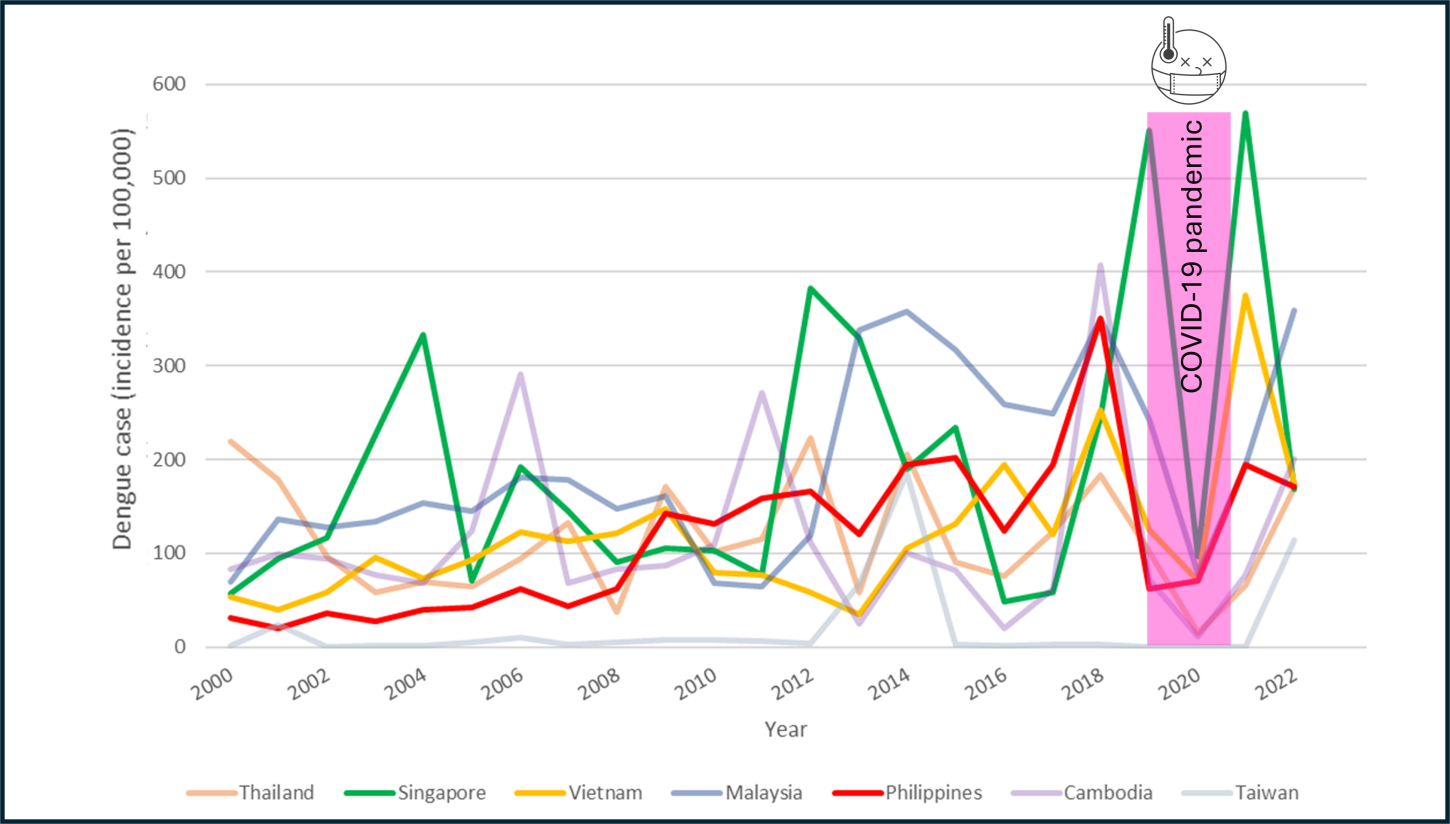
Dynamic trends in dengue incidence in Southeast Asia.

**Figure 2.**
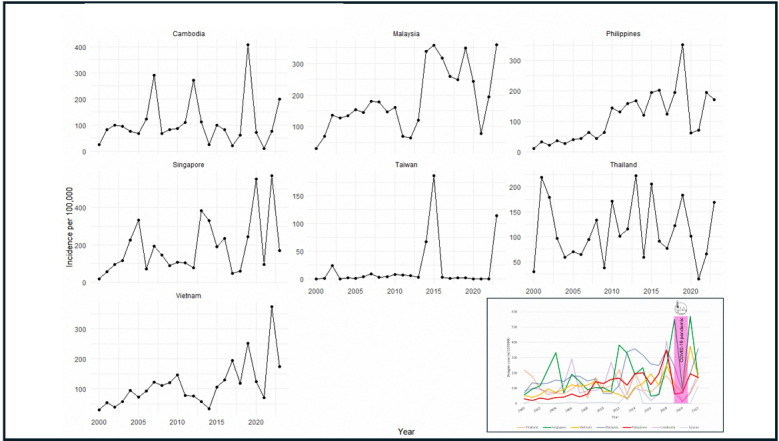
Trends in dengue incidence in individual countries in Southeast Asia. The inset figure illustrates the annual trends of each country and indicates that no multicountry clusters were observed.

After excluding countries with incomplete data, including Laos, Myanmar, Brunei, Indonesia, and Timor-Leste, 7 countries were included in the final analysis: Thailand, Singapore, Vietnam, Malaysia, the Philippines, Cambodia, and Taiwan. A schematic illustration of the included countries is shown in Multimedia Appendix 2. The annual trends in average temperature across these countries over the 24-year study period are presented in Multimedia Appendix 3, demonstrating an overall temperature increase ranging from 0.97 °C to 2.22 °C. The dynamic trends in dengue cases for the countries are plotted in Figure 1 and Multimedia Appendix 4. Fluctuations in dengue incidence were observed across countries, and dengue was more prevalent in Malaysia, Singapore, and Vietnam. A nadir of dengue cases occurred during the COVID-19 pandemic in 2020, and a resurgence of dengue cases was reported after 2022. Dengue incidence in individual countries is plotted in Figure 2; most countries had a rising incidence during the 24 years. There was no multicountry cluster. The peak incidence was approximately 500 cases per 100,000 inhabitants, observed in Singapore. We further examined the linear trend of dengue incidence in each country and summarize the slopes and *R*^2^ values in Multimedia Appendix 5. All slopes were positive, except for Thailand. Significant increasing trends in dengue incidence were identified in Singapore, Vietnam, Malaysia, and the Philippines (slopes: 8.243, 6.513, 8.737, and 8.172; *R*^2^ values: 0.14, 0.34, 0.35, and 0.46, respectively; all *P* values <.05).

**Figure 3.**
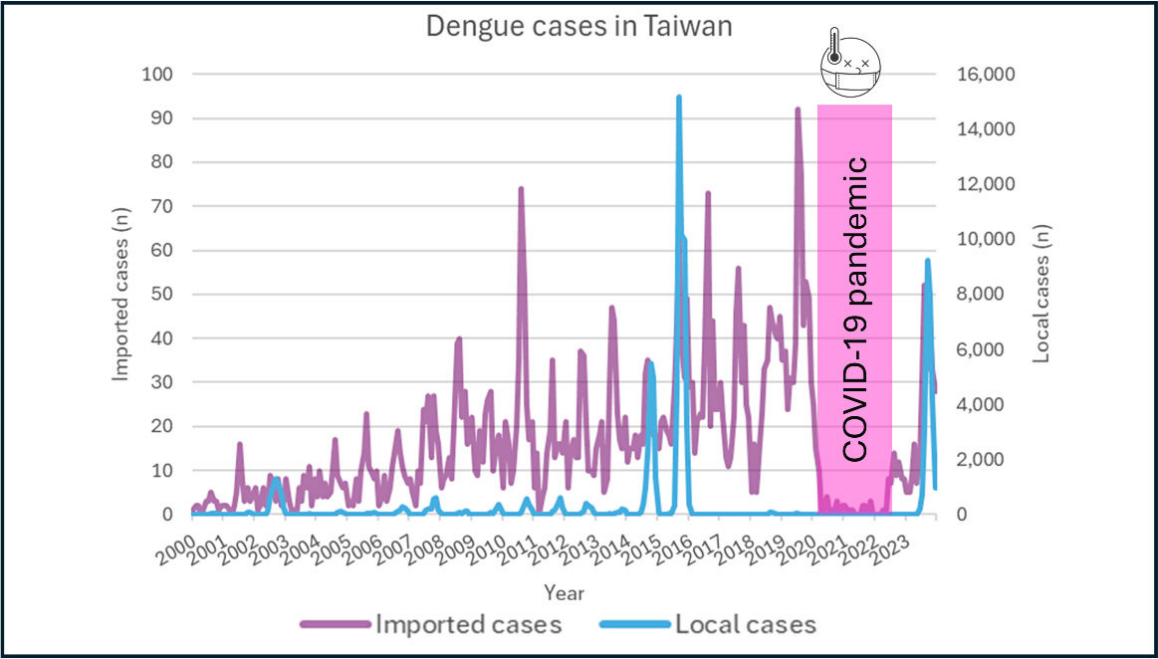
Epidemiological changes in imported and local cases in Taiwan.

The epidemiological trend for imported and local cases in Taiwan is plotted in Figure 3. Sporadic imported cases with an increasing trend were observed before COVID-19 (2000‐2019: slope=0.15; *R*^2^=0.46). A significant surge of local cases was observed in 2014, 2015, and 2023. However, a substantial decline in both imported and local cases was found during the COVID-19 pandemic. A gradual increase in imported cases occurred after the ease of restrictions in 2022, followed by an exponential surge in local cases in 2023.

## Discussion

### Principal Findings

The disease burden of dengue is unquestionably substantial. Our study elucidates the epidemiological changes in an endemic region over a 24-year period. Despite advances in public health, we observed a significant increase in dengue incidence in several Southeast Asian countries, including the Philippines, Vietnam, Malaysia, and Singapore. Other countries demonstrated relatively stable endemic patterns. While dengue cases declined during the COVID-19 pandemic, a notable resurgence was observed across all studied countries, underscoring dengue’s continued significance as a critical public health threat in the postpandemic era.

A marked nadir in dengue incidence was observed during the COVID-19 pandemic. Public health strategies, implementation of nonpharmaceutical interventions, and social and international restrictions likely contributed to this observed reduction. In Sri Lanka, for instance, national dengue case numbers significantly decreased during lockdown periods [31]. In Australia, dengue cases nearly disappeared during the pandemic [32]. Our study revealed that Thailand, Cambodia, and Taiwan reported fewer than 30 cases per 100,000 inhabitants during this period. However, the apparent decrease in reported dengue cases may be attributable to underreporting due to several factors: inadequate medical resources, compromised communication systems, limited diagnostic capabilities, and reduced health care–seeking behavior. Most dengue infections present with mild symptoms such as fever, headache, arthralgia, and skin rashes, and individuals with mild illness may have been less likely to seek medical attention due to reduced health care services and concerns about potential COVID-19 exposure. Additionally, health care providers were primarily focused on COVID-19 diagnostics, potentially leading to decreased dengue screening and testing. During the COVID-19 pandemic, neither the virus nor vector-borne mosquitoes disappeared; instead, they remained latent within communities, waiting for opportunities to resurface. In the aftermath of the COVID-19 pandemic, an “immune debt” phenomenon has emerged, leading to the resurgence of numerous infectious diseases, including influenza, enterovirus, respiratory syncytial virus, and dengue fever. Multiple countries, such as Brazil, Australia, Egypt, and Taiwan, have experienced significant dengue fever outbreaks following the relaxation of pandemic-related restrictions [32-37]. Our research has uncovered similar trends: during the COVID-19 pandemic, stringent preventive measures and reduced international travel disrupted typical epidemiological patterns. Consequently, the health threat posed by dengue fever remains a critical public health concern in the postpandemic era.

Climate change and global warming may contribute to the expanded distribution of vector mosquitoes and increased dengue incidence [11,12,35,38-41]. The optimal temperature for *Aedes albopictus* and *Aedes aegypti* is approximately 25 °C to 30 °C [10]. Warmer temperatures can shorten viral replication incubation periods in mosquitoes, accelerate mosquito growth, and increase biting frequency. Our study showed an overall temperature increase ranging from 0.97 °C to 2.22 °C. Consequently, climate change may expand vector habitat ranges and potentially increase infection transmission zones. Theoretically, dengue is more prevalent in tropical regions. Our study showed a relatively lower incidence in Taiwan, a subtropical country. However, dengue prevalence is influenced by multiple complex factors. We found that the dengue incidence rates significantly increased over the study period in the Philippines, Vietnam, Malaysia, and Singapore. However, these countries were not those with the lowest latitudes or the highest average temperatures among the study sample. Urbanization may reduce breeding sites, alter water accumulation patterns, and impact vector proliferation. Population density and international travel resumption potentially contribute to disease spread [42]. Interestingly, some research has explored correlations between economic indicators like gross domestic product (GDP) and dengue transmission, though findings remain inconsistent [43,44]. Countries with lower GDP often have limited investment in public health infrastructure, medical services, and vector control programs, which may contribute to higher dengue transmission. Conversely, widespread dengue outbreaks may negatively impact national economies by reducing tourism revenue and workforce productivity, thereby further decreasing GDP. In brief, the incidence of dengue epidemics is fueled by multiple complex factors [45,46]. Data from the past 24 years indicate that these evolving trends cannot be explained by any single factor alone.

### Limitations

Our study possesses notable strengths, primarily stemming from its comprehensive analysis of national databases that systematically tracked epidemiological trends across 7 countries over an extensive 24-year period, encompassing both pre- and post–COVID-19 pandemic epochs. However, the research is not without limitations. The study was unable to comprehensively characterize the severity of dengue infections, which range from asymptomatic to severe. Moreover, multiple complex and potentially influential factors remained unaccounted for in this analysis, including nuanced variations in climate patterns, urbanization levels, international travel dynamics, economic indicators, and vaccination status. These unexamined variables could potentially modulate dengue transmission in ways not fully captured by our current methodological approach. Due to the unavailability of reliable data, several countries were excluded from the final analysis. Consequently, while our findings provide valuable insights into regional dengue epidemiology, they also underscore the necessity for future research that can more comprehensively integrate these multifaceted determinants of disease transmission.

### Conclusion

Our 24-year epidemiological analysis of dengue fever in Southeast Asia reveals the complex dynamics of disease transmission. The COVID-19 pandemic temporarily disrupted transmission patterns, but the subsequent resurgence highlights the virus’s resilience. Ongoing, multicountry surveillance efforts are essential for the effective prevention and control of dengue.

## Supplementary material

10.2196/70491 Multimedia Appendix 1 Flowchart of database search and enrollment.

10.2196/70491 Multimedia Appendix 2 Geographic illustration of enrolled countries.

10.2196/70491 Multimedia Appendix 3 Average temperature trends during the study period.

10.2196/70491 Multimedia Appendix 4 Dengue cases and incidences per 100,000 inhabitants in Southeast Asia (2000-2023).

10.2196/70491 Multimedia Appendix 5 The linear trend, slopes, and *R*^2^ values of dengue incidence in each country between 2000 and 2023.

## Abbreviations

GDP: gross domestic product
WHO: World Health Organization
