# Supplementary figures and images for: Dengue Epidemiology in 7 Southeast Asian Countries: 24-Year, Retrospective, Multicountry Ecological Study
Source: Interact J Med Res. 2025 Sep 8;14:e70491. doi: 10.2196/70491 (PMC12416874; doi:10.2196/70491)

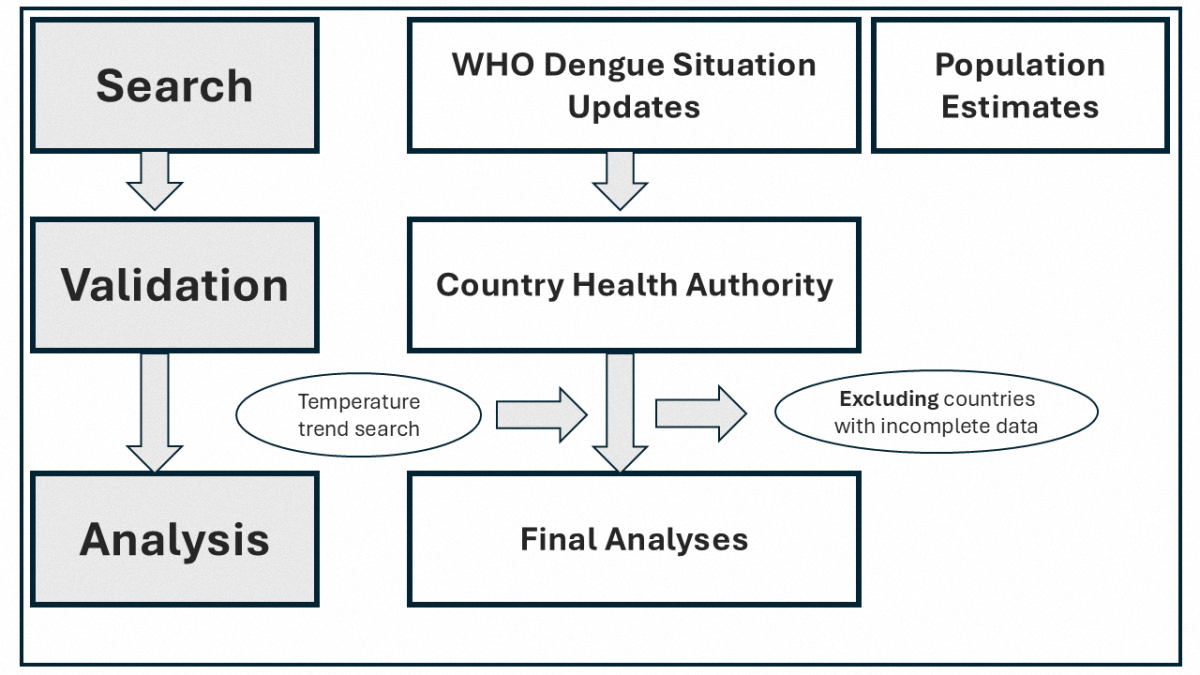

Supplement: Multimedia Appendix 1 [file ijmr-v14-e70491-s001.PNG]

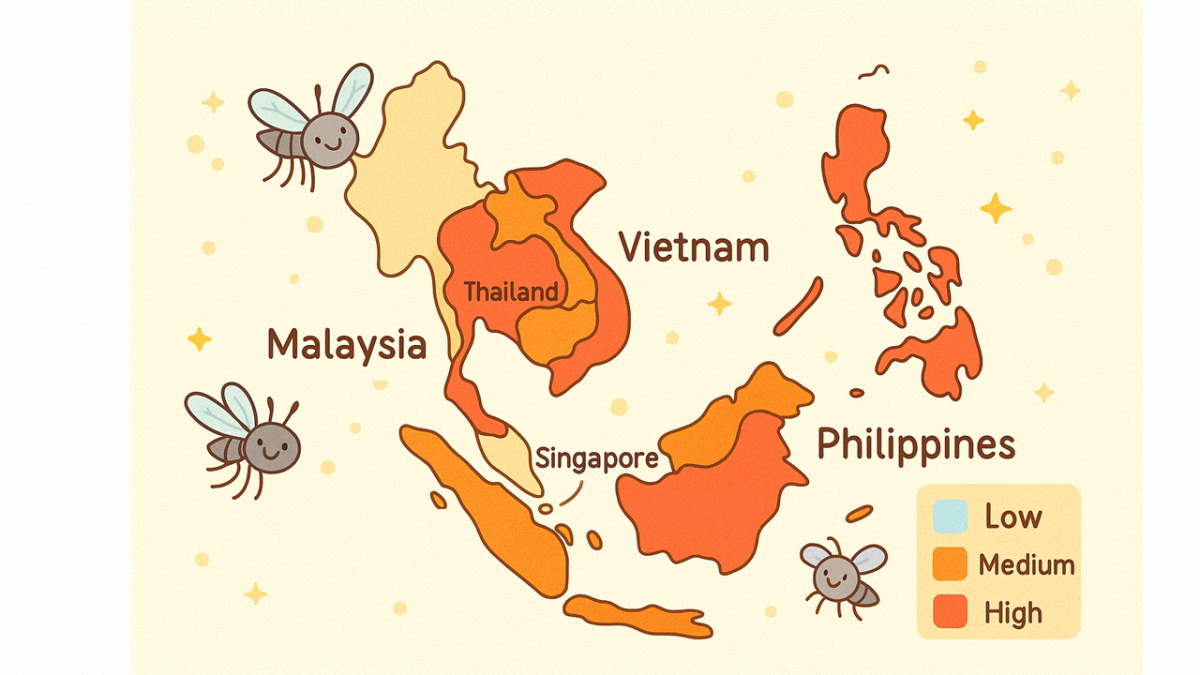

Supplement: Multimedia Appendix 2 [file ijmr-v14-e70491-s002.PNG]

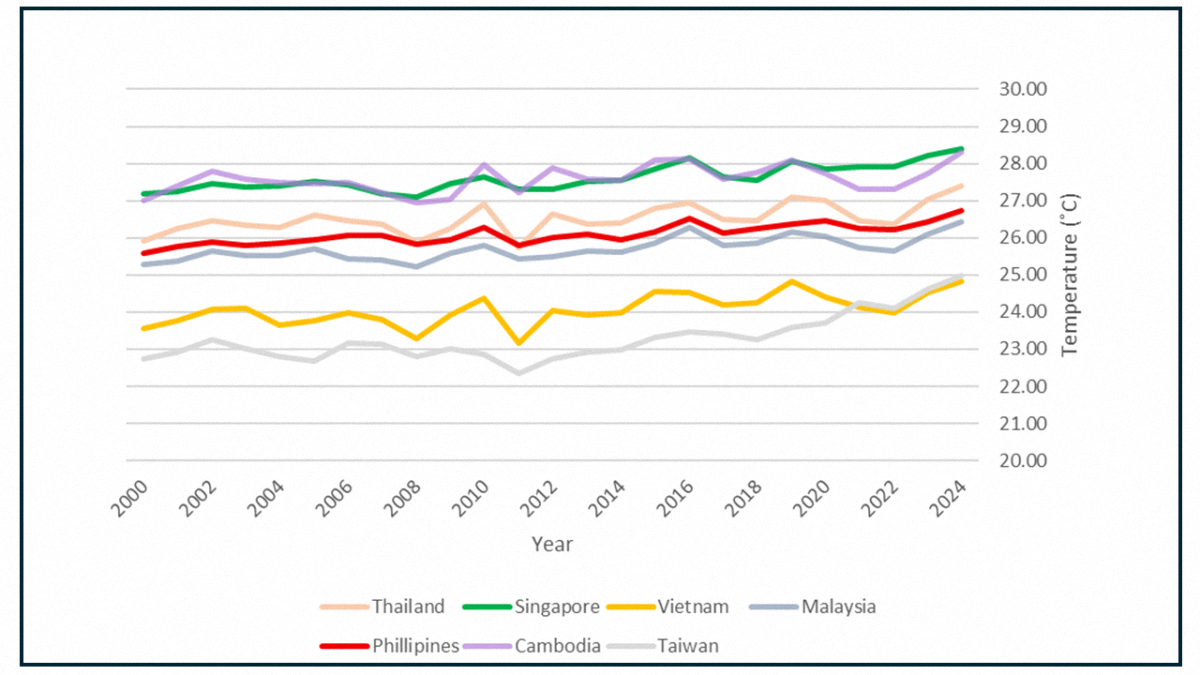

Supplement: Multimedia Appendix 3 [file ijmr-v14-e70491-s003.PNG]
