# Supplementary material for: Dengue Epidemiology in 7 Southeast Asian Countries: 24-Year, Retrospective, Multicountry Ecological Study
Source: Interact J Med Res. 2025 Sep 8;14:e70491. doi: 10.2196/70491 (PMC12416874; doi:10.2196/70491)
Supplement: Multimedia Appendix 4 [file ijmr-v14-e70491-s004.docx]

Table S1. Dengue cases and incidences per 100,000 inhabitants in Southeast Asia (2000-2023)

| Year | Thailand | | Singapore | | Vietnam | | Malaysia | | Philippines | | Cambodia | | Taiwan | |
| --- | --- | --- | --- | --- | --- | --- | --- | --- | --- | --- | --- | --- | --- | --- |
|  | Cases (n) | incidence | Cases (n) | incidence | Cases (n) | incidence | Cases (n) | incidence | Cases (n) | incidence | Cases (n) | incidence | Cases (n) | incidence |
| 2000 | 18617 | 29.5 | 673 | 16.7 | 24116 | 30.5 | 7118 | 31.0 | 8480 | 10.9 | 3148 | 26.0 | 139 | 0.6 |
| 2001 | 139327 | 218.9 | 2372 | 57.3 | 42878 | 53.7 | 16368 | 69.5 | 25216 | 31.7 | 10266 | 83.2 | 281 | 1.3 |
| 2002 | 114800 | 178.8 | 3945 | 94.5 | 31754 | 39.4 | 32767 | 135.7 | 16663 | 20.5 | 12441 | 99.0 | 5388 | 23.9 |
| 2003 | 62767 | 96.9 | 4788 | 116.4 | 47731 | 58.6 | 31545 | 127.5 | 29946 | 36.1 | 12081 | 94.5 | 145 | 0.6 |
| 2004 | 38367 | 58.7 | 9459 | 227.0 | 78692 | 95.6 | 33895 | 133.8 | 23040 | 27.2 | 9983 | 76.7 | 427 | 1.9 |
| 2005 | 45893 | 69.7 | 14209 | 333.1 | 60982 | 73.3 | 39686 | 153.1 | 33901 | 39.3 | 9040 | 68.2 | 306 | 1.3 |
| 2006 | 42456 | 64.0 | 3127 | 71.0 | 77780 | 92.6 | 38556 | 145.4 | 37101 | 42.2 | 16669 | 123.7 | 1074 | 4.7 |
| 2007 | 62949 | 94.2 | 8826 | 192.3 | 104393 | 123.2 | 48846 | 180.3 | 55639 | 62.1 | 39851 | 290.6 | 2179 | 9.5 |
| 2008 | 89626 | 133.1 | 7032 | 145.3 | 96451 | 112.7 | 49335 | 178.3 | 39620 | 43.4 | 9542 | 68.4 | 714 | 3.1 |
| 2009 | 25194 | 37.2 | 4497 | 90.2 | 105370 | 121.8 | 41486 | 147.0 | 57819 | 62.2 | 11699 | 82.6 | 1052 | 4.6 |
| 2010 | 116947 | 171.3 | 5364 | 105.7 | 128831 | 147.4 | 46171 | 160.8 | 135355 | 143.0 | 12500 | 87.0 | 1896 | 8.2 |
| 2011 | 69800 | 101.6 | 5330 | 102.8 | 69680 | 78.9 | 19884 | 68.1 | 125975 | 130.8 | 15980 | 109.6 | 1702 | 7.3 |
| 2012 | 79594 | 115.1 | 4063 | 76.5 | 69023 | 77.3 | 19029 | 64.2 | 154945 | 158.1 | 40164 | 271.6 | 1478 | 6.3 |
| 2013 | 154773 | 222.4 | 20682 | 383.1 | 52913 | 58.6 | 36021 | 119.5 | 166107 | 166.6 | 16722 | 111.5 | 860 | 3.7 |
| 2014 | 41082 | 58.7 | 17992 | 328.9 | 31848 | 34.9 | 103610 | 338.5 | 121580 | 120.0 | 3724 | 24.5 | 15732 | 67.1 |
| 2015 | 144952 | 206.2 | 10470 | 189.2 | 97484 | 105.7 | 111285 | 358.2 | 200415 | 194.5 | 15412 | 100.0 | 43784 | 186.4 |
| 2016 | 63931 | 90.5 | 13091 | 233.5 | 122020 | 131.0 | 100028 | 317.3 | 211108 | 201.3 | 12843 | 82.2 | 744 | 3.2 |
| 2017 | 53961 | 76.1 | 2689 | 47.9 | 183287 | 194.9 | 82840 | 259.1 | 131827 | 123.5 | 3195 | 20.2 | 343 | 1.5 |
| 2018 | 86922 | 122.2 | 3259 | 57.8 | 113850 | 120.0 | 80615 | 248.8 | 211571 | 194.9 | 9885 | 61.7 | 533 | 2.3 |
| 2019 | 131157 | 183.9 | 13951 | 244.6 | 241863 | 252.5 | 114745 | 349.8 | 387254 | 350.8 | 65961 | 407.0 | 640 | 2.7 |
| 2020 | 72578 | 101.5 | 31338 | 551.2 | 121398 | 125.6 | 80590 | 242.7 | 69185 | 61.7 | 11877 | 72.4 | 137 | 0.6 |
| 2021 | 10617 | 14.8 | 5248 | 96.2 | 69354 | 71.2 | 26365 | 78.5 | 79872 | 70.1 | 1903 | 11.5 | 12 | 0.1 |
| 2022 | 46755 | 65.2 | 32130 | 570.0 | 367729 | 374.5 | 66102 | 194.8 | 224477 | 194.3 | 12985 | 77.4 | 88 | 0.4 |
| 2023 | 119965 | 169.4 | 9939 | 168.2 | 172000 | 174.6 | 123133 | 358.8 | 195603 | 170.7 | 35390 | 200.2 | 26706 | 114.0 |

a Footnotes: n= number of cases; incidence= cases per 100,000 inhabitants.
