# Supplementary material for: Dengue Epidemiology in 7 Southeast Asian Countries: 24-Year, Retrospective, Multicountry Ecological Study
Source: Interact J Med Res. 2025 Sep 8;14:e70491. doi: 10.2196/70491 (PMC12416874; doi:10.2196/70491)
Supplement: Multimedia Appendix 5 [file ijmr-v14-e70491-s005.docx]

Table S2. The linear trend, slopes, and R-squared values of dengue incidence in each country between 2000 and 2023

| Country | Thailand | Singapore | Vietnam | Malaysia | Philippines | Cambodia | Taiwan |
| --- | --- | --- | --- | --- | --- | --- | --- |
| 2000 | 29.52 | 16.71 | 30.53 | 31.02 | 10.88 | 25.98 | 0.62 |
| 2023 | 169.42 | 168.19 | 174.63 | 358.75 | 170.69 | 200.19 | 114.03 |
| Slope | -0.7758 | 8.243 | 6.5132 | 8.737 | 8.1723 | 0.9098 | 1.5851 |
| R-squared value | 0.0077 | 0.1402 | 0.3377 | 0.345 | 0.4592 | 0.0044 | 0.0575 |
| *p* | 0.925 | 0.039 | 0.002 | 0.002 | <0.01 | 0.553 | 0.229 |
